# *Acinetobacter baumannii* in Human Body Louse

**DOI:** 10.3201/eid1009.040242

**Published:** 2004-09

**Authors:** Bernard La Scola, Didier Raoult

**Affiliations:** * Faculté de Médecine de Marseille, Marseille, France

**Keywords:** Acinetobacter, Acinetobacter baumanii, louse, homeless, dispatch

## Abstract

While we were isolating *Bartonella quintana* from body lice, 40 *Acinetobacter baumannii* strains were also isolated and genotyped. One clone was unique and the other was ampicillin susceptible. *A. baumannii* DNA was later detected in 21% of 622 lice collected worldwide. These findings show an *A. baumannii* epidemic in human body lice.

The body louse has been demonstrated to be the vector of three human pathogens: *Rickettsia prowazekii*, the agent of epidemic typhus; *Bartonella quintana*, the agent of trench fever; and *Borrelia recurrentis*, the agent of louseborne recurrent fever (*1*). While trying to isolate *Bartonella quintana* from body lice of homeless persons in Marseille, we isolated six *Acinetobacter* spp (*2*). subsequently identified as *A. baumannii*. They were susceptibile to ampicillin, whereas *Acinetobacter* are almost always resistant to ampicillin in France (*3*). We further isolated other *A. baumannii* from body lice in Marseille and now have 40 isolates; 21 are susceptible to ampicillin. To investigate the possibility of a clonal diffusion in lice, the *rec*A gene sequence of isolates was determined and compared to that of the collection and strains. To test if the body louse–*A. baumannii* association is observed worldwide, we investigated the presence of *A. baumannii* DNA in a large collection of body lice.

## The Study

The 40 body lice–associated *A. baumannii* were obtained during studies of homeless shelters in Marseille (*4*). The procedure for isolation of these strains has been detailed previously (*2*). Provisional identification of isolates was based on Gram stain and results of oxidase test and API 20NE identification strip (Biomérieux, Marcy l'Etoile, France). We also tested the 19 strains of *A. baumannii* available at the CIP (Institut Pasteur, Paris, France) and 3 clinical strains isolated in our laboratory during the same period (Table 1). Bacteria were routinely grown at 37°C with 5% CO_2_ on Columbia sheep blood agar (Biomerieux). The *rec*A gene amplification was performed with specific primers rA1 (5´-CCTGAATCTTCTGGTAAAAC-3´) and rA2 (5´-GTTTCTGGGCTGCCAAACATTAC-3´), as described previously (*5*). All sequences were manually edited, and all ambiguous parts were amplified and sequenced again. Amplifying the *rec*A gene allowed unambiguous determination of the sequence of a 336-bp fragment for all isolates. Variation in nucleotides occurred at 10 positions and determined eight genotypes (Table 1), which have been deposited in the GenBank database with the following accession no.: 1, AY274826; 2, AY274827; 3, AY274828; 4, AY274829; 5, AY274830; 6, AY274831; 7, AY274832; 8, AY274833. The translated protein sequences were all identical, except for genotype 2, in which a valine was replaced by an isoleucine at position 68. *rec*A types 1 and 2 were isolated from body louse–associated *A. baumannii*; for the 21 collection strains, seven genotypes were observed. Genotype 2 was unique to body louse–associated *A. baumannii*. Genotype 1 was associated with susceptibility to ampicillin in body louse–associated *A. baumannii* and was common to seven collection strains. However, all collection strains, whatever the genotype, were resistant to ampicillin, even strains of the Unité des Rickettsies that have the same geographic origin as the body louse *A. baumannii*.

**Table 1:** Types of *rec*A gene sequences^a^

*Acinetobacter baumannii* strains (n = 62)	*rec*A type	Ampicillin susceptibility
Lice associated (n = 21)	1	Yes
Lice associated (n = 19)	2	No
CIP 70.34, CIP 70.32, UR 121120, CIP 70.8, CIP 70.9, CIP 70.33, UR 73415	1	No
CIP 54.147, CIP 70.28, CIP 103572, UR 37033, CIP 53.77, CIP 70.22, CIP 105742	3	No
CIP 70.24, CIP 68.38	4	No
CIP 70.10, CIP 70.21	5	No
CIP 54.97	6	No
CIP 53.79	7	No
CIP 64.1	8	No

^a^CIP, strains from the Collection de l'Institut Pasteur (Paris, France); UR, clinical strains from Unité des Rickettsies.

We then tested a large collection of body lice for *A. baumannii* DNA. We tested by polymerase chain reaction (PCR) a collection of 622 body lice sampled in France, Burundi, Rwanda, Peru, Algeria, Portugal, and the Netherlands (*6*). Fifty laboratory lice were used as controls. Detection was performed by amplifying the *rec*A gene with *A. baumannii*–specific primers ACI381F (5´-CACAATGACATTGCAAGCAATTG-3´) and ACI382R (5´-CCAATTTTCATACGAATCTGG-3´) specifically designed for this study. These primers were previously shown not to produce amplicons from *A. calcoaceticus*, *Acinetobacter* genospecies 3, *Acinetobacter* genospecies 13, *A. haemolyticus*, *A. johnsonii*, or *A. lwoffi*. As control for PCR amplification, we used 18Saidg-18Sbi primer pair, which allows amplification of an 18S rRNA gene fragment of arthropods. Consensus forward primer 18Saidg (5´-TCTGGTTGATCCTGCCAGTA-3´) was determined after alignment of 18S rRNA sequences of *Drosophila melanogaster* (GenBank accession no. M21017) and *Aedes aegypti* (GenBank accession no. M95126). The consensus reverse primer 18Sbi primer was the one described by DeSalle et al. (*7*). A total of 130 (21%) body lice were positive for *A. baumannii* (Table 2). None of the 50 laboratory lice was positive. To investigate the genotype association observed among *A. baumannii* strains isolated from Marseille, we sequenced 50 *rec*A amplicons obtained from the lice of different geographic origins (Table 2). Genotypes 1 and 2 were the only ones detected in France; genotype 2 was found in France only. In other parts of the world, genotypes 1, 3, and 4 were observed, with a predominance of genotype 1, similar to the findings in France. Type 4 genotype was the second most common genotype but was absent in European lice. It seems that body louse–associated *A. baumannii* are oligoclonal, and their distribution is different from that of collection strains. Even if genotype 1 is the most common in all cases, genotypes 2 and 4 are overrepresented in body louse–associated strains. However, contrary to culture after body lice decontamination, we cannot rule out that *A. baumannii* infection occurred through external contamination.

**Table 2:** Detection of *Acinetobacter baumannii* in body lice from diverse countries by using *rec*A polymerase chain reaction amplification and sequencing

Country	Body lice tested	Detected *A. baumannii* (%)	Sequenced	*recA* type (no.)
France	340	60 (18)	20	1 (18), 2 (2)
Burundi	88	3 (3)	3	1 (3)
Rwanda	45	26 (58)	10	1 (8), 4 (2)
Peru	60	21 (35)	10	1 (3), 3 (5), 4 (2)
Algeria	54	11 (20)	4	4 (4)
Portugal	10	1 (10)	1	1 (1)
the Netherlands	25	8 (32)	2	1 (2)
All tested	622	130 (21)	50	1 (35), 2 (2), 3 (5), 4 (8)

## Conclusions

The genotype of the 40 *A. baumannii* from Marseille from the body lice of homeless persons are limited to two clones; one is exclusively associated with strains caused by body lice, and the other is associated with ampicillin susceptibility in body louse–associated strains. This finding shows an *A. baumannii* epidemic in body lice. *A. baumannii* is mainly implicated in cases of hospital-acquired infections but has also been reported as a cause of severe community-acquired infections, including pneumonia, endocarditis, and meningitis, mostly in persons who are alcoholics (*8*). While ingesting only blood from humans, the louse has a sterile midgut, and the presence of bacteria is likely caused by the louse's ingesting contaminated blood (*2*). Moreover, previous studies have shown that *A. baumannii* is not a common skin-associated *Acinetobacter* in Europe, unlike in tropical areas, since it is found on the skin of <1.5% of healthy persons (*9*). Our results indicate that association of *A. baumannii* with body lice is likely caused by undiagnosed transient *A. baumannii* bacteremia in patients harboring body lice; however, because the frequency of skin association of *A. baumannii* in the homeless subpopulation is unknown, contamination from body lice cannot be ruled out. Relatively low-virulence flora, such as *Staphylococcus epidermidis* or diptheroids, may be destroyed by leukocytes, antibody, and complement in the blood meal, whereas the more virulent bacteria, such as *A. baumannii*, could survive because they resist the defense mechanism of the blood meal and those of the body lice. However, we never isolated *S. aureus* from lice, which is a virulent bacterium and known to be a common skin commensal agent. From preliminary work, we have observed that body lice may be infected by several bacterial species (L. Houamdi, unpub. data) and that the occurrence of body louse–transmitted disease occurs because causative bacteria (*B. quintana*, *R. prowazekii*, and *B. recurrentis*) induce relapsing bacteremia rather than specifically adapting to body lice (*10*). Finally, if our hypothesis of *A. baumannii* bacteremia in patients harboring body lice is true, their clinical manifestations in homeless persons remain to be determined.

## References

[1] Raoult D, Roux V. The body louse as a vector of reemerging diseases. Clin Infect Dis. 1999;29:888–911. 10.1086/520454 10589908

[2] La Scola B, Fournier PE, Brouqui P, Raoult D. Detection and culture of *Bartonella quintana, Serratia marcescens* and *Acinetobacter* spp. from decontaminated human body lice. J Clin Microbiol. 2001;39:1707–9. 10.1128/JCM.39.5.1707-1709.2001 11325978 PMC88013

[3] Bergogne-Berezin E. *Acinetobacter* species. In: Yu VL, Merigan TC, Barriere SL, editors. Antimicrobial therapy and vaccines. Baltimore: Williams & Wilkins; 1999. p. 3–9.

[4] Brouqui P, La Scola B, Roux V, Raoult D. Chronic *Bartonella quintana* bacteremia in homeless patients. N Engl J Med. 1999;340:184–9. 10.1056/NEJM199901213400303 9895398

[5] Krawczyk B, Lewandowski K, Kur J. Comparative studies of the *Acinetobacter* genus and the species identification method based on the recA sequences. Mol Cell Probes. 2002;16:1–11. 10.1006/mcpr.2001.0388 12005442

[6] Fournier PE, Ndihokubwayo JB, Guidran J, Kelly PJ, Raoult D. Human pathogens in body and head lice. Emerg Infect Dis. 2002;8:1515–8. 12498677 10.3201/eid0812.020111 PMC2738510

[7] DeSalle R, Gatesy J, Wheeler W, Grimaldi D. DNA sequences from a fossil termite in oligo-miocene amber and their phylogenetic implications. Science. 1992;257:1933–6. 10.1126/science.1411508 1411508

[8] Chen MZ, Hsueh PR, Lee LN, Yu CJ, Yang PC, Luh KT. Severe community-acquired pneumonia due to *Acinetobacter baumannii.* Chest. 2001;120:1072–7. 10.1378/chest.120.4.1072 11591541

[9] Berlau J, Aucken H, Malnick H, Pitt T. Distribution of *Acinetobacter* species on skin of healthy humans. Eur J Clin Microbiol Infect Dis. 1999;18:179–83. 10.1007/s100960050254 10357050

[10] Chu YW, Leung CM, Houang ET, Ng KC, Leung CB, Leung HY, Skin carriage of acinetobacters in Hong Kong. J Clin Microbiol. 1999;37:2962–7. 10449482 10.1128/jcm.37.9.2962-2967.1999 PMC85423

